# Improvement of cake baking properties by lipases compared to a traditional emulsifier

**DOI:** 10.1016/j.fochx.2022.100442

**Published:** 2022-09-09

**Authors:** Charlotte Dorothea Stemler, Katharina Anne Scherf

**Affiliations:** Department of Bioactive and Functional Food Chemistry, Institute of Applied Biosciences, Karlsruhe Institute of Technology (KIT), Adenauerring 20 a, Karlsruhe 76131, Germany

**Keywords:** Enzymes, Lipids, Mono- and diacetyl tartaric acid esters of mono- and diglycerides of fatty acids (DATEM), Product quality, Shelf-life, Texture

## Abstract

Lipases are commonly used as clean-label improvers for bread. However, their potential use in cakes with different formulations remains unknown. The aim was to analyze the effects of seven baking lipases on three different cake formulations (an eggless cake, a pound cake with eggs and a yeast-based cake) in comparison to a traditional emulsifier. Product density, water loss during baking and product texture were assessed. If and to what extent the product quality was improved depended on both the lipase and the cake formulation. Lipase-induced effects mostly exceeded those of the emulsifier and were most pronounced in formulations without intrinsic emulsifiers like eggs. The lipases differed in their extent of improvement, hinting at the importance of their specific reactivity patterns and the resulting range of interactions with macromolecules. Further research is needed to unravel the mechanistic background of baking quality improvement in cakes.

## Highlights

• Lipases can improve the baking characteristics of different cakes.
• In comparison to DATEM they lead to softer products and less staling.
• The use of eggs or yeast diminishes the improvement by lipases.
• Lipase activity and specificity influence the extent of improvement.

## Graphical abstract

**Figure:**
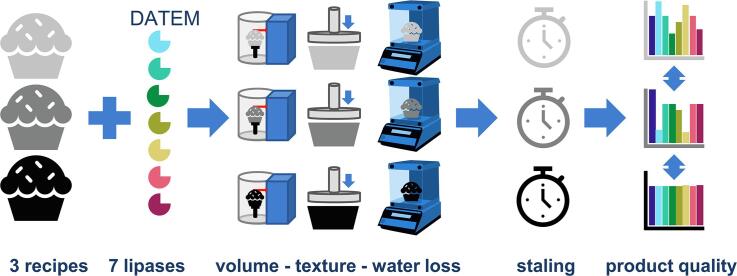

## 1 Introduction

Lipases are enzymes that catalyze the hydrolysis of lipids and they have been used since 1990 as baking improvers (Ramrakhiani & Chand, 2011). Their effects on the product quality of bread are comparable or even greater than the ones of traditional emulsifiers like mono- and diacetyl tartaric acid esters of mono- and diglycerides of fatty acids (DATEM, E472e) (Moayedallaie et al., 2010, Colakoglu and Özkaya, 2012). Increased product volume and reduced firmness are among the described improvements (Moayedallaie et al., 2010, Schaffarczyk et al., 2014, Gerits et al., 2015) as well as an inhibition of staling (Stojceska and Ainsworth, 2008, Gerits et al., 2015, Purhagen et al., 2011). The effects are due to the reaction of lipases within bread dough that creates more polar lipids, for example by hydrolysis of triacylglycerols to di- and monoacyglycerols. As summarized by Gerits et al. (2014a), those polar lipids then cause the described product quality improvements mainly by three different mechanisms of action: (i) interactions with the gluten network, (ii) stabilization of gas cells in the dough and (iii) interactions with starch.

Concerning the gluten network, polar lipids enhance gluten aggregation by decreasing the electrostatic repulsion between gluten polymers and thereby increase gluten network strength (Koehler, 2001). Gluten quantity and quality and the resulting gluten network strength are decisive for the baking quality of wheat flour and can even be used to predict bread volumes (Geisslitz et al., 2018, Wieser et al., 2022). A strengthened gluten network also indirectly affects gas cell stability (Pareyt et al., 2011). Additionally, gas cell stability and gas cell formation are directly affected by the incorporation of polar lipids into the gas-liquid-interface of dough (Gan et al., 1995, Sroan and MacRitchie, 2009). Gas cells are formed when air is incorporated into the liquid phase of the dough upon agitation. They act as nucleation sites for CO_2_ and enable oven spring by bubble expansion. The ability to maintain bubble integrity is crucial for the textural and sensory attributes of bread (Mills et al., 2003). If the gas cells stay intact during dough expansion and baking, they lead to light and even crumb textures (Gan et al., 1995). Polar lipids are known to improve the ability of the liquid films to maintain bubble integrity (Gan et al., 1995). When polar lipids interact with starch, they reduce both initial firmness and the extent of staling. The former is due to the formation of amylose-lipid complexes after addition of lipases (Stojceska & Ainsworth, 2008), the latter to the reduction of the extent of amylopectin retrogradation by lipase reaction products such as free fatty acids and “lyso”lipids (Purhagen et al., 2011, Gerits et al., 2015).

It is therefore desirable to transfer this application of baking lipases to cakes. Cakes had a global market size of USD 42.94 billion in 2019 and an estimated compound annual growth rate of 3.3 % until 2027 (Grand View Research, 2019). The use of baking lipases as a replacement for traditional emulsifiers could lower production costs and enable the production of “clean label” products, because lipases are inactivated during baking (Gerits et al., 2014a).

However, only little is known about the use of baking lipases in cakes. One first study suggests a possible transferability of the effects known from bread to cake: Guy and Sahi showed that the produced surfactants stabilized gas bubbles in cake dough, leading to a finer texture and a greater extension of the products. Staling effects as measured by eating quality and perceived freshness were also improved (Guy & Sahi, 2006). However, they only used one lipase and one cake recipe. An in-depth analysis of the functionality of different lipases in cakes is missing. No broad-range industrial applications for baking lipases in cakes have been established, either. This is mostly due to two factors. First, off-flavors can occur when lipases react with high amounts of fat as used in cakes. This is especially the case when butter is part of the recipe and short-chain fatty acids like butyric acid are released. This problem can be overcome by the use of lipases which specifically do not release short-chain fatty acids. Second, while bread mostly consists of flour, water, yeast and small amounts of salt and sugar, there are many different definitions for cake in different parts of the world. Their ingredient functionality differs depending on the recipe. Most include flour, sugar, eggs, fats or oils and leavening agents. The formation of a gluten network is no longer crucial for baking quality. Instead, fat, sugar and eggs all act as structural components (Wilderjans et al., 2013). Besides varying functional ingredients, also the broad range of lipid classes from added fat and eggs hinder the prediction of the effect of lipases on cake product quality.

The hypothesis is that different baking lipases differently affect the product quality of cakes, depending on the recipe. Our aim was therefore to analyze the impact of a range of baking lipases on different cake recipes in comparison to the traditional emulsifier DATEM. DATEM was chosen because it is one of the most widely applied emulsifiers in the baking industry (Moayedallaie et al., 2010). It has been used before in comparative studies for the use of lipases in bread (Moayedallaie et al., 2010, Colakoglu and Özkaya, 2012) and its mechanisms of action are well known (Koehler, 2001). Three different cake recipes were chosen: first a basic cake without eggs (BC), second a traditional pound cake (PC) with eggs and third brioche, a yeast-based cake. Product density, water loss during baking and product texture were measured. Staling was recorded as change of textural properties over 72 h. The novelty of our work is the analysis of the effects of several lipases in different cake recipes compared to DATEM. In this way the knowledge about baking lipase reactions in cake will be deepened, paving the way for promising strategies to replace DATEM and for further research in this field.

## 2 Materials and methods

### 2.1 Reagents and ingredients

All chemical reagents were of analytical grade or higher and the glassware used was grade A volumetric glassware only. Wheat flour (Type 405, GoodMills GmbH, Hamburg, Germany) and extra-white powdered sugar (Nordzucker, Braunschweig, Germany), both of commercial quality, were kindly donated by Dr. August Oetker Nahrungsmittel KG (Bielefeld, Germany). Pasteurized whole eggs, baking powder, butter (82 % fat) and fresh yeast were purchased at a local supermarket. DATEM was kindly donated by backaldrin The Kornspitz Company (Asten, Austria).

### 2.2 Lipases

After preliminary tests, seven commercial baking lipases were chosen according to their substrate specificity: They were shown to release no short-chain fatty acids using the *p–*nitrophenyl assay (Stemler & Scherf, 2022). All are known for their improvement of the baking characteristics of bread. No off-flavors caused by the lipases were detected in the samples by an untrained sensory panel consisting of 12 panelists (data not shown). The seven lipases used were from Kerry Group (Tralee, Ireland), Novozymes (Bagsværd, Denmark), ABEnzymes (Darmstadt, Germany) and DSM (Heerlen, The Netherlands) and kindly donated by Kuchenmeister (Soest, Germany), DSM, ABEnzymes and Novozymes. The specific types of lipases are given in Table 1. They are randomly named lipase A, E, G, J, K, M and O.

**Table 1:** Lipases, dosage and lipase activity measured using the three different lipase activity assay kits MAK046, MAK047 and MAK048. Values are given as mean (n = 3) ± standard deviation.

Lipase	Type of lipase	Dosage		Lipase activity		
		Brioche, based on flour	Basic cake, pound cake, based on batter	MAK 046	MAK 047	MAK 048
		[mg kg^−1^]	[mg kg^−1^]	[µmol min^−1^mg^−1^]	[nmol min^−1^mg^−1^]	[nmol min^−1^mg^−1^]
A	Phospho	120	400	1.78 ± 0.12	63.87 ± 1.13	1427.38 ± 21.95
E	Phospho	150	500	0.57 ± 0.04	23.30 ± 0.32	1306.23 ± 8.55
G	Unknown	90	300	2.05 ± 0.08	121.99 ± 2.57	2003.26 ± 158.15
J	Phospho	20	70	1.82 ± 0.04	201.96 ± 3.21	2244.74 ± 81.07
K	Glyco	90	300	0.64 ± 0.04	6.68 ± 0.14	162.78 ± 12.42
M	Phospho	60	200	1.06 ± 0.08	60.96 ± 0.73	1490.79 ± 69.54
O	TAG	60	200	0.27 ± 0.02	68.84 ± 2.30	861.91 ± 70.54

Glyco; glycolipase; Phospho, phospholipase; TAG, triacylglycerol lipase.

### 2.3 Lipase activity assays

The three commercially available lipase activity assay kits MAK046, MAK047 and MAK048 (Merck KGaA, Darmstadt, Germany) were used for lipase activity measurements. The lipases were dissolved at a concentration of 1 mg mL^−1^ in buffer (50 mmol L^−1^ Tris-HCl, pH 7.5, 1 mmol L^−1^ CaCl_2_) (Glogauer et al., 2011). For the lipases A, G and J in MAK047, further dilution 1:10 (v:v) in lipase buffer was necessary. For MAK048, all lipase solutions were further diluted 1:100 (v:v). All tests were carried out in triplicate according to the manufacturer’s instructions. A Tecan multiplate reader Infinite 200 Pro (Tecan Group, Männedorf, Switzerland) was used for measurement of absorbance at 570 nm for MAK046 and at 412 nm for MAK047 or fluorescence for MAK048 (excitation at 529 nm and emission at 600 nm). After measurement, the linear range of absorption or fluorescence change over time was manually double-checked and adjusted in a way that the coefficient of determination was ≥ 0.95 before calculating lipase activities. This procedure was applied to avoid negative lipase activities as occurring when the calculation was performed according to the manufacturer’s instructions (data not shown).

### 2.4 Cake preparation

The cakes were prepared in a commercial food processor with planetary mixing (Robert Bosch GmbH, Stuttgart, Germany) equipped with a whisk and a kneading hook. Preliminary tests were performed with the lipase dosages as suggested by the manufacturers (data not shown). As only slight improvements were partly visible, the dosages were doubled. The final lipase dosages are given in Table 1.

To avoid weighing errors, the lipases were dissolved in water and added by volume. An equal amount of water was added for the control sample without lipase addition. The DATEM sample was prepared accordingly, by adding 112 mg of DATEM (BC and PC) or 180 mg of DATEM (brioche), as DATEM does not dissolve in water. Additional incubation times for the reaction of lipases with the batters/doughs were added in the recipes as suggested by Gerits et al. (Gerits et al., 2014b) (for details see 2.4.1 to 2.4.3). Eight muffins with a batter/dough weight of 50 g were prepared from each modified batter/dough, proofed for 20 min at 37 °C and baked in a preheated hot air oven (UNOX Deutschland GmbH, Büren, Germany) at 180 °C for 12 min.

#### 2.4.1 Basic cake

For BC, 100 g of butter, 50 g of sugar and 2.5 g of salt were mixed for 2.5 min. Then, 200 mL of water, 250 g of flour and 15 g of baking powder were added and mixed for another 3 min. Aliquots of 400 g of batter were thoroughly mixed for 30 s with lipases, DATEM or water (control) and incubated for 1 h at room temperature in plastic boxes before baking.

#### 2.4.2 Pound cake

For PC, 200 g of butter, 200 g of sugar and 2 g of salt were mixed for 3 min. Pasteurized eggs (200 g) were added and blended in for another 3 min. Finally, 200 g of wheat flour and 0.6 g of baking powder were added and mixed in for 3 min. As described for BC, aliquots of 400 g of batter were mixed with or without additives and incubated for 1 h at room temperature in plastic boxes before baking.

#### 2.4.3 Brioche

For brioche, 300 g of flour, 125 mL of pre-heated water (37 °C), 35 g of yeast and 50 g of pasteurized egg were thoroughly kneaded for 10 min. Then, aliquots of 240 g of dough were mixed with water, lipase solution, or water and DATEM and left to proof for 2 h at 37 °C in a proofing cabinet in open plastic boxes. After proofing, 50 g of butter, 20 g of sugar, 80 g of flour and 2 g of salt were added to each aliquot and the mixture was kneaded for another 5 min.

### 2.5 Product characteristics

Water loss during baking, muffin density and texture were assessed. Each test was performed six times, measuring two muffins each from one batter/dough. The remaining muffins were stored in closed ziplock bags at room temperature until measurement.

Water loss of the muffins during baking was calculated by comparing the weight of the batter/dough of a sample and the weight of the resulting muffin after cooling for 2 h.

Muffin volumes were measured by the use of a VolScan Profiler VSP300 (Stable Micro Systems, Godalming, Surrey, UK) with a rotation speed of 0.5 s^−1^ and a vertical step size of 1 mm. The quotient of muffin weight and muffin volume was used as muffin density.

Textural characteristics of the muffins were measured directly after cooling (0 h) and 24 h, 48 h and 96 h after baking. The tops of the muffins were removed with a knife and the remaining muffin slices with a height of 3 cm were immediately used for texture profile analysis (TPA). TPA was performed using a TA.XTplus texture analyzer (Stable Micro Systems) equipped with a heavy duty platform and a 20 mm diameter cylindrical probe. The following settings were used for the double compression test: pretest speed 1 mm s^−1^, test speed and backtest speed 0.8 mm s^−1^, deformation to 40 % of the sample height, 10 s waiting time between the measurements, a release force of 0.049 N and a measurement data rate of 200 measuring points per second. The software Exponent (version 6.1.16.0, by Stable Micro Systems) was used for data evaluation and the instrument was calibrated regularly using a test weight, according to the instructions of the manufacturer. As preset by the software, product firmness is defined as the maximum peak height of the first compression. Springiness is calculated as the quotient of the time needed for the first compression and the time needed for the second compression. Cohesiveness is the peak area of the second compression divided by the peak area of the first compression. The quotient of the peak area of the first compression before the maximum peak height and the remaining peak area of the first peak is defined as resilience. Additionally, the two dependent parameters gumminess (firmness multiplied by cohesiveness) and chewiness (gumminess multiplied by springiness) were calculated. A typical curve of a TPA of cake with further explanation of the calculation of textural properties is given in Fig. S1.

### 2.6 Statistical analysis

Origin 2021b was used for statistics (OriginLab Corporation, Northampton, MA). Means and standard deviations were calculated for each value. An analysis of variance (ANOVA) with Tukey’s test (p ≤ 0.05) was used to detect significant differences between the means of all values at a certain time. Additional ANOVA tests with two-sided Dunnett’s t-tests (p ≤ 0.05) were performed in IBM SPSS Statistics 27 (International Business Machines Corporation, Armonk, NY, USA) to detect significant differences of a modified sample to the control sample at the respective time.

## 3 Results

### 3.1 Lipase activities

The lipases chosen for analysis comprised four phospholipases, one glycolipase, one triacylglycerol lipase and one with unknown specificity (Table 1). The activities measured by the three different assays are not directly comparable, because the assays rely on different reaction mechanisms. From what is known from the chemicals included, MAK046 presumably relies on the release of glycerol from triolein, MAK047 likely monitors lipase reactions with a thioester and MAK048 allegedly measures the hydrolysis of a bond between glycerol and glutaric acid (Beisson et al., 2000, Hasan et al., 2009).

The overall ranges for the seven lipase activities lay between 0.27 and 2.05 µmol min^−1^ mg^−1^ (MAK046), 6.68 and 201.96 nmol min^−1^ mg^−1^ (MAK047) as well as 162.78 and 2244.74 nmol min^−1^ mg^−1^(MAK048), showing an up to 32-fold range within one assay (Table 1). The lipases G and J had comparably high activities in all three assays while the lipases E and K reacted rather slowly. The lipases A and M ranged in between. The activity of lipase O was dependent on the chosen assay. While it had the lowest activity of all lipases in MAK046, it reacted strongly with the thioester used for analysis in MAK047. There it displayed the third–highest activity of all lipases, ranging directly after the lipases J and G. In MAK048 however, lipase O again had a comparably low activity.

### 3.2 Basic cake

**Fig. 1:**
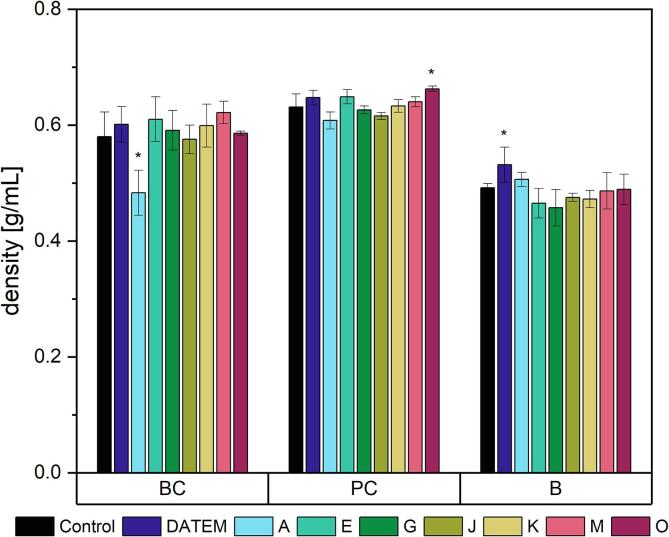
Density of differently modified basic cake (BC), pound cake (PC) and brioche (B) samples (Control: sample without lipase addition, DATEM: sample with addition of DATEM, A-O: samples with addition of the respective lipase). Asterisks show a significant difference to the control (ANOVA with Dunnett’s test, p ≤ 0.05, n = 6).

Several product characteristics of BC were improved by the use of baking lipases. BC density ranged from 0.48 to 0.62 g mL^−1^ with an average of 0.58 g mL^−1^ (Fig. 1, Table S1). Only one lipase led to a significant reduction of density by 17 % compared to the control (0.48 g mL^−1^ instead of 0.58 g mL^−1^). No significant change in product density occurred for the remaining six lipases and for DATEM.

**Fig. 2:**
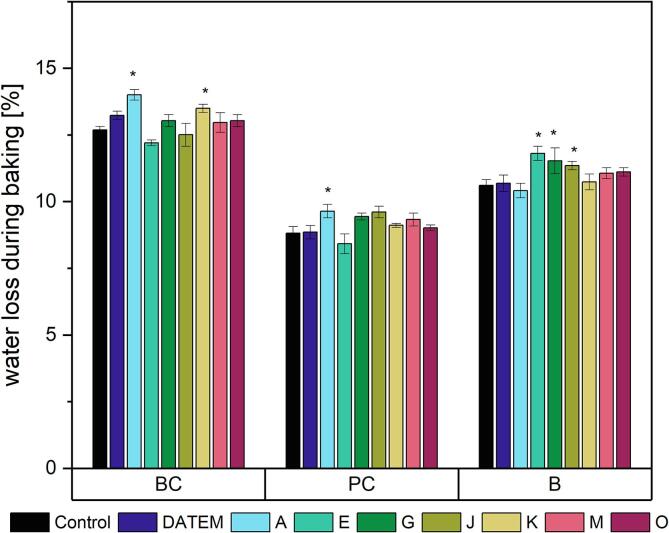
Water loss during baking of differently modified basic cake (BC), pound cake (PC) and brioche (B) samples (Control: sample without lipase addition, DATEM: sample with addition of DATEM, A-O: samples with addition of the respective lipase). Asterisks show a significant difference to the control (ANOVA with Dunnett’s test, p ≤ 0.05, n = 6).

Two lipases affected the water loss of BC significantly (Fig. 2, Table S1). Both lipases A and K caused a significant increase in water loss compared to the control sample (10.5 % and 6.5 %, respectively).

**Fig. 3:**
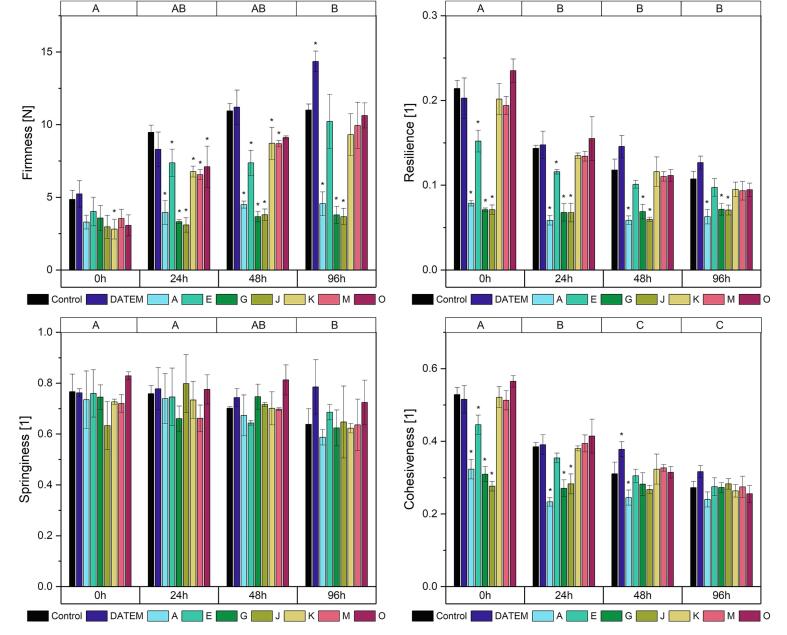
Firmness, resilience, springiness and cohesiveness after 0 h, 24 h, 48 h and 96 h of differently modified basic cake samples (Control: sample without lipase addition, DATEM: sample with addition of DATEM, A-O: samples with addition of the respective lipase). Asterisks show a significant difference to the control sample of the respective time (ANOVA with Dunnett’s test, p ≤ 0.05, n = 6). Capital letters on the top indicate significant differences between means of all values of a certain time (ANOVA with Tukey’s test, p ≤ 0.05, n = 6).

BC firmness showed an overall increase due to staling between the measurements directly after baking and after 96 h (Fig. 3, Table S1). The firmness of the control sample increased from 4.9 N to 11.0 N. The initial firmness was only slightly affected by the addition of lipases. Only lipase K led to a reduction of initial firmness by 42 % compared to the control. During storage, product firmness was affected by more lipases: after 24 h, all seven lipases led to a significant improvement of firmness ranging from 22.2 % (lipase E) to 67.3 % (lipase J) compared to the control. Especially lipases A, G and J were highly effective. A similar trend was observed after 48 h, where lipases A, G and J reduced the firmness by 58.9 %, 66.5 % and 65.3 % compared to the control, respectively. Lipases E, K and M also had a significant impact on product firmness but only to a maximum extent of 32.7 % (lipase E). After 96 h, only the three most effective lipases A, G and J still led to significantly softer products. While the initial firmness of the control sample had increased by 120.0 %, the firmness of the samples treated with these three lipases increased only by 38.4 % (lipase A), 13.6 % (lipase G) and 23.9 % (lipase J). DATEM had no effect on BC product firmness.

The resilience of BC showed an overall decrease after the first day of storage and it remained stable afterwards (Fig. 3, Table S2). Again, the lipases A, G and J had the highest impact. Concerning the initial resilience, also lipase E affected the resilience, albeit to a smaller extent: directly after baking, lipase A reduced the resilience by 63.3 %, lipase G by 66.8 %, lipase J by 66.8 % and lipase E by 29.0 % compared to the control. The extent of reduction became smaller during the storage time. After 96 h, it was 41.5 % for lipase A, 33.4 % for lipase G and 34.2 % for lipase J. Still, only those three lipases led to significant changes compared to the control for all measurements of resilience. Again, no significant improvement was observed when DATEM was used (p = 0.16 to 0.79).

BC springiness was reduced overall after storage times between 24 h and 96 h from an average springiness of all samples from 0.74 to 0.66 (Fig. 3, Table S3). Neither DATEM nor the lipases affected BC springiness significantly at any time.

Overall, BC cohesiveness decreased over time (Fig. 3, Table S3). Significant changes were visible between the measurements directly after baking and after 24 h and 48 h. The highest effects by lipases occurred directly after baking, when the lipases A, E, G and J caused a significant reduction of cohesiveness with a maximum of 47.6 % by lipase J. After 24 h of storage, only the effects caused by the lipases A, G and J were still significant. After 48 h, lipase A was the only one leading to a significant decrease in cohesiveness. DATEM on the other hand negatively affected product cohesiveness and led to its significant increase. After 96 h, no significant changes occurred.

As expected from the effects on firmness and cohesiveness, the two dependent parameters gumminess and chewiness (Fig. S2, Table S4) were positively affected by lipase addition, with the highest extent caused by the lipases A, G and J.

### 3.3 Pound cake

All analyzed product characteristics of PC were affected by the addition of lipases. PC product density ranged from 0.61 to 0.66 g mL^−1^ with an average of 0.64 g mL^−1^ (Fig. 1, Table S1). It was only slightly negatively affected by the addition of lipase O, which caused an increase of 5.0 % compared to the density of the control.

Concerning the water loss during baking of PC samples, a slight increase (9.6 % instead of 8.8 % for the control) occurred when lipase A was applied (Fig. 2, Table S1).

**Fig. 4:**
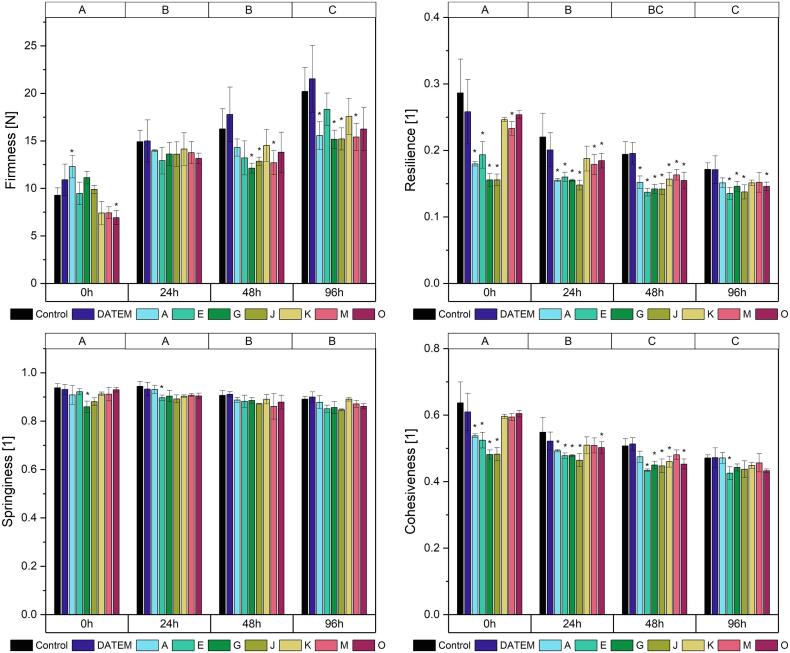
Firmness, resilience, springiness and cohesiveness after 0 h, 24 h, 48 h and 96 h of differently modified pound cake samples (Control: sample without lipase addition, DATEM: sample with addition of DATEM, A-O: samples with addition of the respective lipase). Asterisks show a significant difference to the control sample of the respective time (ANOVA with Dunnett’s test, p ≤ 0.05, n = 6). Capital letters on the top indicate significant differences between means of all values of a certain time (ANOVA with Tukey’s test, p ≤ 0.05, n = 6).

PC product texture was affected in various ways. Its firmness was partly reduced by lipase addition (Fig. 4, Table S1). PC samples became firmer with storage, showing significant differences between all samples measured directly after baking (mean: 9.4 N) and 24 h afterwards (mean: 14.0 N), as well as between the samples measured 48 h (mean: 14.2 N) and 96 h after baking (mean: 17.3 N). Several lipases reduced the staling of the samples and had the greatest impact on the firmness after 96 h of storage (reduction of firmness by 23.0 %, 24.9 %, 24.7 % and 23.8 % by the lipases A, G, J and M, respectively). In comparison to those values, only three lipases softened the products after 48 h of storage (lipase G by 25.4 %, lipase J by 21.0 % and lipase M by 21.9 %) and none led to significant differences to the control after 24 h of storage. Directly after baking, only lipase O exerted softening effects on the samples (firmness reduction of 25.5 %), while lipase A even led to a firmer product (32.6 %). As already seen for BC, DATEM had no effect on product firmness.

Concerning PC resilience (Fig. 4, Table S2), the lipases caused a reduction and its extent decreased with storage time. The overall product resilience also decreased with storage time, showing significant differences between all values measured directly after baking and 24 h afterwards, as well as between the storage for 48 h and 96 h. Directly after baking, the lipases A, E, G, J and M significantly reduced the resilience compared to the control sample with a maximum of 45.7 % (lipase G). During storage, the maximum effects were a reduction to 32.9 % (lipase J) after 24 h, 30.0 % (lipase J) after 48 h and 21.0 % (lipase E) after 96 h. Again, no significant alterations of resilience occurred when DATEM was applied to the sample.

The overall springiness of PC decreased significantly during storage for 24 h and 48 h (Fig. 4, Table S3). It was only slightly affected by lipases, the only significant changes being a reduction of 8.4 % directly after baking by lipase G and a reduction of 5.0 % after 24 h of storage by lipase E compared to the control. DATEM exerted no effect on PC springiness.

Similar to PC resilience, its overall cohesiveness decreased during storage and the greatest impact of lipase addition occurred directly after baking (Fig. 4, Table S3). Before storage, PC cohesiveness was reduced by a maximum of 32.0 % (lipase J), less after 24 h (17.6 %, lipase J) and 48 h (15.4 %, lipase E) and only 9.7 % of reduction (lipase E) remained after 96 h of storage. DATEM did not lead to any significant changes in PC cohesiveness.

As PC firmness and cohesiveness were affected by lipase addition, the dependent parameters gumminess and chewiness were also improved, whereby chewiness was affected to a greater extent (Fig. S3, Table S4).

### 3.4 Brioche

The product characteristics of brioche were affected to a smaller extent by the addition of lipases than the ones of BC and PC. The mean density of brioche samples was 0.49 g mL^−1^, ranging from 0.46 to 0.53 g mL^−1^ (Fig. 1, Table S1). It was only affected by the addition of DATEM, which led to an increase of density by 8.2 % compared to the control.

The average water loss of brioche during baking was 11.0 % (Fig. 2, Table S1). It was significantly increased compared to the control sample by the addition of the lipases E, G and J (11.3 %, 8.7 % and 7.0 %, respectively).

**Fig. 5:**
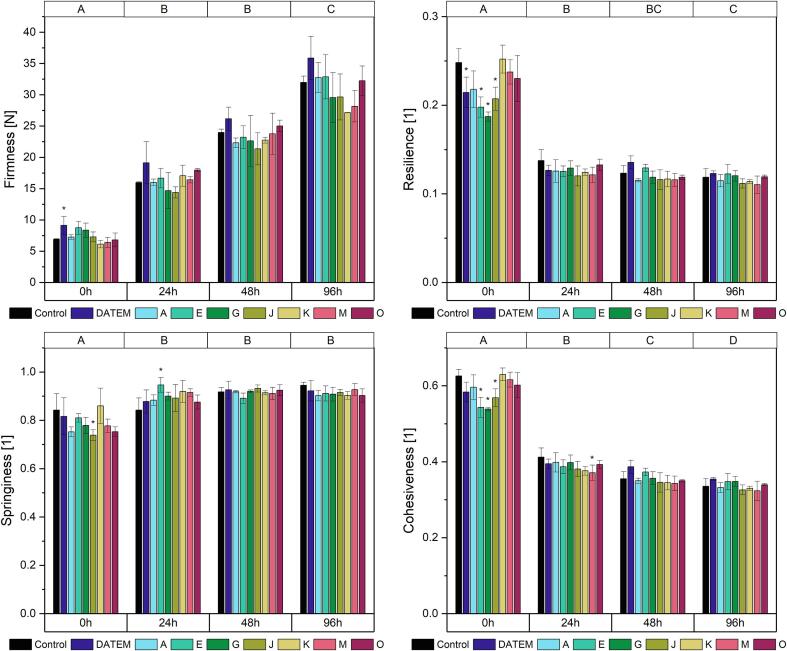
Firmness, resilience, springiness and cohesiveness after 0 h, 24 h, 48 h and 96 h of differently modified brioche samples (Control: sample without lipase addition, DATEM: sample with addition of DATEM, A-O: samples with addition of the respective lipase). Asterisks show a significant difference to the control sample of the respective time (ANOVA with Dunnett’s test, p ≤ 0.05, n = 6). Capital letters on the top indicate significant differences between means of all values of a certain time (ANOVA with Tukey’s test, p ≤ 0.05, n = 6).

The firmness of brioche samples increased significantly over time, starting with a mean firmness of 7.5 N directly after baking to 16.5 N after 24 h, 23.5 N after 48 h and to 31.1 N after 96 h of storage (Fig. 5, Table S1). The addition of lipases did not affect the firmness significantly. DATEM led to a 32.0 % increase of initial firmness directly after baking, but had no effects during storage of the samples.

As described for BC and PC, the overall product resilience of brioche decreased over time (Fig. 5, Table S2). Significant differences were detected between the mean resilience measured directly after baking and after 24 h of storage as well as between the ones 24 h after baking and 96 h after baking. Effects caused by the added improvers were only detectable directly after baking, where DATEM and the lipases E, G and J led to reductions of 13.6 %, 23.1 %, 30.8 % and 21.8 % of control sample resilience, respectively.

Contrary to the results in BC and PC, the springiness of brioche increased during storage (Fig. 5, Table S3). In two cases the added improvers exerted significant effects on the springiness: Directly after baking, lipase J caused a 12.3 % reduction compared to the control sample and 24 h after baking; lipase E led to a 12.4 % increase.

Concerning the cohesiveness of brioche, a steady increase during storage could be observed (Fig. 5, Table S3). The mean cohesiveness changed from 0.59 directly after baking to 0.39 after 24 h, 0.36 after 48 h and 0.34 after 96 h of storage. Significant effects of lipase addition occurred directly after baking (reductions of 13.2 %, 13.9 % and 9.2 % by the lipases E, G and J compared to the control sample, respectively) and 24 h after baking (reduction of 10.0 % by lipase M). Further changes of brioche cohesiveness were not significant.

The dependent parameters gumminess and chewiness of brioche were not affected by the addition of improvers (Fig. S4, Table S4).

## 4 Discussion

The lipase activities were comparatively similar for the lipases A, E, G, J, K and M in all assays. Lipase O showed a strong dependency on the substrate used. Those results are, however, not consistent with the dosage recommendations for the lipases. Lipase G, for example, is to be used in the same concentration as lipase K, although lipase G ranged among the lipases with the highest activities in all assays and K among the ones with the lowest activities. This is in accordance with the results from Gerits et al. (2014b) who stated that the *p–*nitrophenyl assay, another artificial assay to assess lipase activity, is not a suitable predictor for the baking performance of lipases. Currently there are no ready-to-use assays to assess the effectiveness of lipases towards those substrates linked to baking performance. Additionally, studies about the lipid classes responsible for the baking quality of cakes are missing. The results were therefore handled with care when discussing the extent of effects on the baking quality of cakes.

The density of cakes is one marker for their baking quality. The addition of baking improvers should lead to a reduction of product density, in line with larger product volumes. Density was, however, only slightly affected by the addition of lipases or improvers in all cakes. Instead of reducing the density as known for their use in bread (Moayedallaie et al., 2010), it partly even led to an increase in PC and brioche. One possible reason might be that the ingredients in bread and cake differ, thus leading to other molecules defining product characteristics. While bread typically consists of flour, salt, yeast, sugar as a starter for the yeast and water (Schaffarczyk et al., 2014), cakes are made of flour, sugar, eggs, fat or oil and leavening agents (Wilderjans et al., 2013). The addition of more surface-active particles might not further improve gas cell stability in cakes as those are already completely covered by suitable layers including proteins or lipids. Similar effects were also observed by Rodríguez-García et al. (2014) who could not identify an improvement of cake density by the addition of a lipase to their cake recipe including egg. Yet, Guy and Sahi (2006) observed significant improvements in cake density working with a different lipase in a cake with a similar recipe. As the ingredients can therefore not be the cause, it is most possibly due to the lipases and their reaction patterns. As in our case also DATEM failed to reduce the density, it might even be possible that surfactants per se cannot improve this marker for baking quality in the studied cakes.

The second marker for baking quality analyzed was the water loss of the products during baking. Similar to the product density, the lipases exerted only slightly negative effects on water loss. When used in bread, lipases did not lead to significant changes (Purhagen et al., 2011). The water loss of products during baking is linked to the water-binding capacity of the ingredients (Aydogdu et al., 2018). Although most lipases did not alter the water loss during baking, this is the first time that lipases were shown to negatively impact the water-binding capacity of bakery goods.

The main marker for baking quality taken into consideration for this study was product texture including firmness, resilience, springiness, cohesiveness, gumminess and chewiness. The greatest changes of product texture occurred in BC. Especially the three lipases A, G and J were highly effective concerning the inhibition of staling and the reduction of resilience and cohesiveness. Their effects were up to three times higher than the ones by other lipases. Similar differences between the effects of different lipases have not been reported previously. Instead, different lipases led to corresponding decreases of firmness and staling in bread (Moayedallaie et al., 2010). Besides the lipases used, this might be due to the broader range of lipid classes available for hydrolysis. As the lipases were developed for use in bread, their interactions with the high amounts of triacylglycerols and phospholipids as well as with sphingolipids and cholesterol, both of which are not present in wheat, are unknown. Possibly their specific interaction patterns and substrate specificities led to larger differences in their performance in cakes compared to bread. Additionally, their lipase activity might be a possible reason: the lipases G and J are the two lipases with the highest activities, thus expected to hydrolyze high amounts of lipids. Lipase A was recommended to be added in comparatively high amounts compared to its activity. All three were also among the lipases with the biggest effects on the texture of PC. The broader range of substrates for lipase reactions than in bread seem to emphasize the importance of suitable reactivity patterns of baking lipases. This hypothesis is further supported by the fact that lipase O, the only triacylglycerol lipase, showed the poorest performance in all measurements. Phospholipases, on the other hand, showed potential for use in cakes.

BC firmness was most improved during storage. An overall reduction of firmness and stiffening during storage by the addition of lipases is known for bread (Gerits et al., 2015, Stojceska and Ainsworth, 2008, Purhagen et al., 2011) and has been shown in cake (Guy & Sahi, 2006). Rodríguez-García et al. (2014), however, did not observe a softening of cakes when lipases were added, although they worked with a similar recipe as Guy and Sahi (2006). While the initial bread firmness is mostly linked to amylose crystallization during cooling (Gerits et al., 2015, Purhagen et al., 2011), long-term firmness is due to amylopectin retrogradation and water migration from gluten to starch (Gerits et al., 2015). Both mechanisms also take place in cake as shown, for example, for the complexation of amylose by lipids in eggless cakes (Lin et al., 2017). Initial firmness in cake has been linked to the number of air bubbles and their uniform distribution in cakes (Rodríguez-García et al., 2014, Psimouli and Oreopoulou, 2013). Air bubbles in cake are mostly affected by fat and, if applicable, egg proteins (Wilderjans et al., 2013). BC does not contain eggs as part of the recipe. Therefore, mostly the interaction of lipase reaction products with amylose and the effects of lipases on the lipid coats of air bubbles are most likely responsible for the change of initial firmness. However, those effects only occurred to a significant extent when the glycolipase K was used. All other lipases performed better during storage and inhibited staling. Hydrolyzed non-glycolipids therefore seemed to only interact with amylopectin, not with amylose.

In PC, the effects of lipase addition on the firmness were similar, albeit to a lesser extent. The lipases A, G and J led to softer cakes after storage, to the same extent as did lipase M. Similar to BC, the initial firmness was only affected by a single lipase (but lipase O instead of lipase K). The extent of softening by lipase reaction products is probably linked to the cake recipe. In contrast to BC, PC contains about 25 % of eggs. 20 % of egg lipids are phosphatidylcholines, also known as lecithins (Belitz et al., 2007). Lecithins are known for their excellent emulsifying properties. Therefore, the addition of more surface-active molecules by the reaction of lipases might not affect the gas bubbles as strongly as it did for BC, where no additional emulsifiers were present.

The complete lack of softening effects of lipases on brioche was rather unexpected. Besides the ingredients used with the lipases in the trials with BC and PC, only yeast was added. Yeast is also present in bread and does not inhibit lipase reactions there. Lipases were added at smaller amounts but were given longer reaction times. A total lack of effects by lipases happened before in trials with lipases in bread (Melis et al., 2019, Schaffarczyk et al., 2014). Explanatory hypotheses given by the authors were that lipases are only active during mixing, but not during fermentation because of the structural organization of their substrates in oil bodies (Melis et al., 2019) or a possible shear sensitivity of lipases leading to their inactivation by mechanical input during mixing (Schaffarczyk et al., 2014). In combination with the smaller amounts of lipases used for brioche in comparison to the other cakes, both could explain the results. Another factor causing their inactivation could be the various fermentation products such as ethanol and succinic acid, which themselves are known to negatively impact baking properties of bread (Jayaram et al., 2014a, Jayaram et al., 2014b).

In contrast to the softening effects for DATEM known from its application in bread (Moayedallaie et al., 2010), it did not influence the firmness of cakes. Lipases are therefore even more suitable than DATEM as additives in this context.

Resilience describes the ability of products to recover after a compression (Psimouli & Oreopoulou, 2013). The resilience of freshly baked bread was shown to depend on both the amylose network formed during cooling and the thermoset gluten network formed during baking (Goesaert et al., 2009). For bread, it has been shown that lipases lead to a reduced resilience, but also flatten the decrease during storage (Gerits et al., 2015). Lipase effects on the resilience of cakes have not been assessed before. In all three analyzed recipes, the initial resilience was reduced when lipases were added. As it was shown for the influence of lipases on the initial firmness, they only scarcely interacted with amylose. Therefore, the observed effects are most probably due to their influence on the gluten network. In BC the reduction of resilience was comparably higher than in PC: Again, the eggs included in PC might be a possible cause for their behaviour. Wilderjans et al. (2010) highlighted the importance of the protein network consisting of egg proteins and gluten for pound cake quality. This cross-linked network possibly reacts differently from a simple gluten network when exposed to polar lipids.

The springiness of all cakes was only slightly affected by the addition of lipases. It is a measure of the recovering ability of the products between two compressions (Psimouli & Oreopoulou, 2013). Springiness has been linked to the strength of interactions between starch and protein fractions of flour in cakes (Rodríguez-García et al., 2014) and is known to decrease during storage (Purhagen et al., 2011). In previous studies focusing on cakes, springiness was decreased by lipase addition (Rodríguez-García et al., 2014); in bread it was stable although softening effects caused by lipases occurred (Purhagen et al., 2011). The analyzed combinations of cakes and lipases did not show trends of springiness enhancement or reduction compared to the control samples.

The cohesiveness describes how the work needed to compress the sample for the first time changes compared to a second compression. It therefore indicates how the internal resistance of the product is damaged when compressed. The cohesiveness of all samples behaved similarly to their resilience: The overall value decreased during storage and lipases had a diminishing effect which was strongest directly after baking. Also, the extent of reduction was the biggest in BC, smaller in PC and smallest in brioche. The underlying mechanisms therefore seem to be similar to the ones for resilience.

## 5 Conclusions

The influence of seven lipases on the baking quality of three different cake recipes was assessed in comparison to the traditional emulsifier DATEM. If and to what extent the baking quality, especially the texture, was improved depended on both the lipase and the recipe. The extent of improvement in BC, an eggless cake, was greater than the one in PC, a cake with eggs, as expected taking into consideration the intrinsic emulsifying properties of eggs. In the yeast-based brioche recipe, only few improvements of baking quality occurred. It is not clear whether this is due to missing activity of the lipases in this system. In BC, especially the lipases A, G and J strongly influenced the baking quality, probably due to their specific reaction patterns. All lipases performed better than DATEM.

Our hypothesis that the extent of baking quality improvement for cakes depends on both the used lipase and the cake formulation was hereby confirmed. The study provides a first overview of possible uses of bread baking lipases in cakes. In terms of possible substitution of traditional emulsifiers, this is especially valuable as common emulsifiers have recently been shown to alter human gut microbiota (Naimi et al., 2021). However, the mechanistic backgrounds of the observed effects are still unclear. The effects of lipases on the various ingredients of cakes have not yet been studied. Especially the requirements concerning the lipid classes they interact with remain unknown. A first step could be an in-depth analysis of the effects on different lipid classes in cakes in combination with the resulting effects on baking quality.

## Declaration of Competing Interest

The authors declare that they have no known competing financial interests or personal relationships that could have appeared to influence the work reported in this paper.
