# Supplementary material for: Improvement of cake baking properties by lipases compared to a traditional emulsifier
Source: Food Chem X. 2022 Sep 9;15:100442. doi: 10.1016/j.fochx.2022.100442 (PMC9532760; doi:10.1016/j.fochx.2022.100442)
Supplement: Supplementary data 1 [file mmc1.pdf]

SUPPLEMENTARY MATERIAL

Improvement of cake baking properties by lipases compared to a traditional emulsifier

Charlotte Dorothea STEMLER[1], Katharina Anne SCHERF[1]*

[1] Department of Bioactive and Functional Food Chemistry, Institute of Applied Biosciences, Karlsruhe Institute of Technology (KIT), Adenauerring 20 a, 76131 Karlsruhe, Germany

Correspondence: Katharina Anne Scherf, Karlsruhe Institute of Technology (KIT), Adenauerring 20 a, 76131 Karlsruhe, Germany, Email: katharina.scherf@kit.edu, Tel.: +49 721 608 42929, https://bioactivefc.iab.kit.edu

ORCID for Charlotte D. Stemler: 0000-0002-6470-1258

ORCID for Katharina A. Scherf: 0000-0001-8315-5400

Table S1. Density, water loss and firmness after 0 h, 24 h, 48 h and 96 h of differently modified basic cake, pound cake and brioche samples. Control: sample without lipase addition, DATEM: sample with addition of DATEM, A-O: samples with addition of the respective lipase. Values are given as mean (n = 6) ± standard deviation.

		Density [g/mL]			Water loss [%]			Firmness [N]											
								0 h			24 h			48 h			96 h		
Basic cake	Control	0.58	±	0.04	12.68	±	0.14	4.87	±	0.62	9.47	±	0.50	10.94	±	0.52	11.01	±	0.41
	DATEM	0.60	±	0.03	13.23	±	0.16	5.23	±	0.91	8.31	±	1.18	11.20	±	1.18	14.35	±	0.71
	A	0.48	±	0.04	14.01	±	0.20	3.29	±	0.47	3.96	±	0.82	4.50	±	0.24	4.56	±	0.82
	E	0.61	±	0.04	12.20	±	0.11	4.02	±	0.99	7.37	±	0.94	7.370	±	0.86	10.23	±	1.85
	G	0.59	±	0.03	13.03	±	0.22	3.34	±	0.76	3.33	±	0.14	3.67	±	0.36	3.79	±	0.59
	J	0.58	±	0.03	12.51	±	0.43	2.97	±	0.79	3.10	±	0.49	3.79	±	0.40	3.67	±	0.57
	K	0.60	±	0.04	13.50	±	0.15	2.81	±	0.68	6.77	±	0.38	8.71	±	1.11	9.31	±	1.44
	M	0.62	±	0.02	12.96	±	0.37	3.54	±	0.64	6.56	±	0.34	8.69	±	0.22	9.95	±	1.59
	O	0.59	±	0.00	13.03	±	0.22	3.07	±	0.73	7.10	±	1.41	9.12	±	0.10	10.63	±	0.87
Pound cake	Control	0.63	±	0.02	8.81	±	0.25	9.27	±	0.77	14.91	±	1.20	16.26	±	2.13	20.20	±	2.55
	DATEM	0.65	±	0.01	8.86	±	0.25	10.91	±	1.65	14.99	±	2.21	17.78	±	2.87	21.54	±	3.51
	A	0.61	±	0.02	9.64	±	0.25	12.30	±	1.20	13.98	±	0.11	14.29	±	0.90	15.56	±	1.48
	E	0.65	±	0.01	8.42	±	0.37	9.46	±	1.19	12.92	±	1.41	13.21	±	1.79	18.31	±	1.72
	G	0.63	±	0.01	9.44	±	0.13	11.13	±	0.67	13.61	±	1.23	12.13	±	0.52	15.17	±	0.96
	J	0.62	±	0.01	9.61	±	0.22	9.90	±	0.42	13.60	±	1.31	12.85	±	0.43	15.21	±	1.16
	K	0.63	±	0.01	9.11	±	0.08	7.40	±	1.25	14.14	±	1.71	14.53	±	1.67	17.58	±	1.90
	M	0.64	±	0.01	9.33	±	0.24	7.43	±	0.63	13.76	±	1.15	12.70	±	1.27	15.40	±	1.44
	O	0.66	±	0.01	9.02	±	0.11	6.91	±	0.74	13.16	±	0.55	13.80	±	2.11	16.24	±	2.25
Brioche	Control	0.49	±	0.01	10.61	±	0.22	6.92	±	0.08	15.98	±	0.12	23.97	±	0.54	31.99	±	1.01
	DATEM	0.53	±	0.03	10.69	±	0.31	9.13	±	1.42	19.13	±	3.37	26.16	±	1.84	35.88	±	3.47
	A	0.51	±	0.01	10.41	±	0.27	7.26	±	0.37	16.01	±	0.51	22.32	±	0.77	32.75	±	2.41
	E	0.47	±	0.03	11.81	±	0.27	8.74	±	1.04	16.67	±	1.56	23.21	±	1.82	32.88	±	3.53
	G	0.46	±	0.03	11.53	±	0.49	8.35	±	1.15	14.68	±	2.87	22.63	±	4.07	29.56	±	3.99
	J	0.48	±	0.01	11.35	±	0.16	7.28	±	0.80	14.39	±	0.89	21.36	±	2.57	29.65	±	3.68
	K	0.47	±	0.02	10.74	±	0.30	6.13	±	0.60	17.06	±	1.68	22.74	±	0.47	27.15	±	0.04
	M	0.49	±	0.03	11.06	±	0.21	6.41	±	0.81	16.41	±	0.50	23.75	±	3.29	28.17	±	2.51
	O	0.49	±	0.03	11.11	±	0.16	6.81	±	1.10	17.98	±	0.22	25.01	±	0.94	32.25	±	2.36

Table S2. Resilience after 0 h, 24 h, 48 h and 96 h of differently modified basic cake, pound cake and brioche samples.
Control: sample without lipase addition, DATEM: sample with addition of DATEM, A-O: samples with addition of the respective lipase. Values are given as mean (n = 6) ± standard deviation.

		Resilience											
		0 h			24 h			48 h			96 h		
Basic cake	Control	0.21	±	0.01	0.14	±	0.00	0.12	±	0.01	0.11	±	0.01
	DATEM	0.20	±	0.02	0.15	±	0.02	0.15	±	0.01	0.13	±	0.01
	A	0.08	±	0.00	0.06	±	0.01	0.06	±	0.01	0.06	±	0.01
	E	0.15	±	0.01	0.12	±	0.00	0.10	±	0.00	0.10	±	0.01
	G	0.07	±	0.00	0.07	±	0.01	0.07	±	0.01	0.07	±	0.01
	J	0.07	±	0.01	0.07	±	0.01	0.06	±	0.00	0.07	±	0.01
	K	0.20	±	0.02	0.13	±	0.00	0.12	±	0.02	0.10	±	0.01
	M	0.19	±	0.01	0.13	±	0.01	0.11	±	0.01	0.09	±	0.01
	O	0.24	±	0.01	0.16	±	0.03	0.11	±	0.01	0.09	±	0.01
Pound cake	Control	0.29	±	0.05	0.22	±	0.04	0.19	±	0.02	0.17	±	0.01
	DATEM	0.26	±	0.05	0.20	±	0.03	0.20	±	0.02	0.17	±	0.02
	A	0.18	±	0.00	0.16	±	0.00	0.15	±	0.01	0.15	±	0.01
	E	0.19	±	0.02	0.16	±	0.01	0.14	±	0.01	0.14	±	0.01
	G	0.16	±	0.01	0.16	±	0.00	0.14	±	0.01	0.15	±	0.01
	J	0.16	±	0.01	0.15	±	0.01	0.14	±	0.01	0.14	±	0.01
	K	0.25	±	0.00	0.19	±	0.02	0.16	±	0.01	0.15	±	0.00
	M	0.23	±	0.01	0.18	±	0.02	0.16	±	0.01	0.15	±	0.01
	O	0.25	±	0.01	0.18	±	0.01	0.15	±	0.01	0.15	±	0.01
Brioche	Control	0.25	±	0.02	0.14	±	0.01	0.12	±	0.01	0.12	±	0.01
	DATEM	0.21	±	0.02	0.13	±	0.01	0.14	±	0.01	0.12	±	0.00
	A	0.22	±	0.02	0.13	±	0.01	0.12	±	0.00	0.11	±	0.01
	E	0.20	±	0.01	0.13	±	0.01	0.13	±	0.00	0.12	±	0.01
	G	0.19	±	0.01	0.13	±	0.01	0.12	±	0.01	0.12	±	0.01
	J	0.21	±	0.01	0.12	±	0.01	0.12	±	0.01	0.11	±	0.01
	K	0.25	±	0.02	0.12	±	0.00	0.12	±	0.01	0.11	±	0.00
	M	0.24	±	0.01	0.12	±	0.01	0.12	±	0.01	0.11	±	0.01
	O	0.23	±	0.03	0.13	±	0.01	0.12	±	0.00	0.12	±	0.00

Table S3. Springiness and cohesiveness after 0 h, 24 h, 48 h and 96 h of differently modified basic cake, pound cake and brioche samples. Control: sample without lipase addition, DATEM: sample with addition of DATEM, A-O: samples with addition of the respective lipase. Values are given as mean (n = 6) ± standard deviation.

		Springiness				Cohesiveness			
		0 h	24 h	48 h	96 h	0 h	24 h	48 h	96 h
Basic cake	Control	0.77 ± 0.07	0.76 ± 0.03	0.70 ± 0.01	0.64 ± 0.06	0.53 ± 0.02	0.38 ± 0.01	0.31 ± 0.03	0.27 ± 0.02
	DATEM	0.76 ± 0.02	0.78 ± 0.08	0.74 ± 0.04	0.79 ± 0.11	0.52 ± 0.04	0.39 ± 0.03	0.38 ± 0.02	0.32 ± 0.02
	A	0.74 ± 0.11	0.74 ± 0.10	0.67 ± 0.08	0.59 ± 0.03	0.32 ± 0.03	0.23 ± 0.01	0.24 ± 0.02	0.24 ± 0.02
	E	0.76 ± 0.09	0.75 ± 0.11	0.64 ± 0.01	0.69 ± 0.03	0.45 ± 0.03	0.35 ± 0.01	0.31 ± 0.02	0.27 ± 0.02
	G	0.74 ± 0.05	0.66 ± 0.05	0.75 ± 0.05	0.62 ± 0.07	0.31 ± 0.02	0.27 ± 0.02	0.28 ± 0.03	0.27 ± 0.01
	J	0.63 ± 0.09	0.80 ± 0.11	0.72 ± 0.01	0.65 ± 0.14	0.28 ± 0.01	0.28 ± 0.03	0.27 ± 0.01	0.28 ± 0.01
	K	0.73 ± 0.01	0.73 ± 0.07	0.70 ± 0.06	0.62 ± 0.02	0.52 ± 0.03	0.38 ± 0.01	0.32 ± 0.04	0.26 ± 0.02
	M	0.72 ± 0.03	0.66 ± 0.05	0.70 ± 0.01	0.64 ± 0.10	0.51 ± 0.03	0.39 ± 0.02	0.33 ± 0.01	0.27 ± 0.03
	O	0.83 ± 0.02	0.78 ± 0.06	0.81 ± 0.06	0.72 ± 0.09	0.57 ± 0.02	0.41 ± 0.05	0.31 ± 0.02	0.26 ± 0.02
Pound cake	Control	0.94 ± 0.02	0.94 ± 0.02	0.91 ± 0.02	0.89 ± 0.01	0.64 ± 0.06	0.55 ± 0.04	0.51 ± 0.02	0.47 ± 0.01
	DATEM	0.93 ± 0.02	0.93 ± 0.03	0.91 ± 0.01	0.90 ± 0.02	0.61 ± 0.06	0.52 ± 0.03	0.51 ± 0.02	0.47 ± 0.03
	A	0.91 ± 0.04	0.93 ± 0.02	0.89 ± 0.01	0.88 ± 0.03	0.54 ± 0.01	0.49 ± 0.00	0.47 ± 0.02	0.47 ± 0.02
	E	0.92 ± 0.01	0.90 ± 0.01	0.88 ± 0.03	0.85 ± 0.01	0.52 ± 0.02	0.48 ± 0.01	0.43 ± 0.00	0.43 ± 0.02
	G	0.86 ± 0.02	0.90 ± 0.02	0.89 ± 0.01	0.86 ± 0.03	0.48 ± 0.01	0.48 ± 0.00	0.45 ± 0.01	0.44 ± 0.01
	J	0.88 ± 0.02	0.89 ± 0.02	0.87 ± 0.00	0.85 ± 0.00	0.48 ± 0.02	0.46 ± 0.02	0.45 ± 0.02	0.44 ± 0.03
	K	0.91 ± 0.01	0.90 ± 0.01	0.89 ± 0.02	0.89 ± 0.01	0.60 ± 0.01	0.51 ± 0.02	0.46 ± 0.02	0.45 ± 0.01
	M	0.91 ± 0.03	0.91 ± 0.01	0.86 ± 0.05	0.87 ± 0.01	0.59 ± 0.01	0.51 ± 0.02	0.48 ± 0.01	0.46 ± 0.03
	O	0.93 ± 0.01	0.90 ± 0.01	0.88 ± 0.03	0.86 ± 0.01	0.60 ± 0.01	0.50 ± 0.02	0.45 ± 0.02	0.43 ± 0.01
Brioche	Control	0.84 ± 0.07	0.84 ± 0.05	0.92 ± 0.02	0.94 ± 0.01	0.63 ± 0.02	0.41 ± 0.02	0.36 ± 0.02	0.34 ± 0.02
	DATEM	0.82 ± 0.08	0.88 ± 0.05	0.93 ± 0.04	0.92 ± 0.04	0.58 ± 0.03	0.39 ± 0.01	0.39 ± 0.02	0.35 ± 0.00
	A	0.75 ± 0.02	0.88 ± 0.02	0.92 ± 0.00	0.90 ± 0.02	0.60 ± 0.03	0.40 ± 0.03	0.35 ± 0.01	0.33 ± 0.01
	E	0.81 ± 0.02	0.95 ± 0.03	0.89 ± 0.02	0.91 ± 0.03	0.54 ± 0.03	0.39 ± 0.02	0.37 ± 0.01	0.35 ± 0.02
	G	0.78 ± 0.03	0.90 ± 0.02	0.92 ± 0.01	0.91 ± 0.03	0.54 ± 0.00	0.40 ± 0.02	0.36 ± 0.02	0.35 ± 0.01
	J	0.74 ± 0.02	0.89 ± 0.06	0.93 ± 0.01	0.92 ± 0.01	0.57 ± 0.02	0.38 ± 0.02	0.35 ± 0.03	0.33 ± 0.01
	K	0.86 ± 0.07	0.92 ± 0.05	0.91 ± 0.01	0.90 ± 0.02	0.63 ± 0.02	0.38 ± 0.01	0.35 ± 0.02	0.33 ± 0.01
	M	0.78 ± 0.03	0.92 ± 0.02	0.91 ± 0.03	0.93 ± 0.02	0.62 ± 0.02	0.37 ± 0.02	0.34 ± 0.02	0.32 ± 0.03
	O	0.75 ± 0.02	0.88 ± 0.03	0.92 ± 0.02	0.90 ± 0.03	0.60 ± 0.03	0.39 ± 0.01	0.35 ± 0.00	0.34 ± 0.00

Table S4. Gumminess and chewiness after 0 h, 24 h, 48 h and 96 h of differently modified basic cake, pound cake and brioche samples. Control: sample without lipase addition, DATEM: sample with addition of DATEM, A-O: samples with addition of the respective lipase. Values are given as mean (n = 6) ± standard deviation.

		Gumminess [N]				Chewiness [N]			
		0 h	24 h	48 h	96 h	0 h	24 h	48 h	96 h
Basic cake	Control	2.55 ± 0.48	3.63 ± 0.15	3.42 ± 0.50	3.00 ± 0.28	1.94 ± 0.27	2.77 ± 0.11	2.44 ± 0.38	1.93 ± 0.34
	DATEM	2.69 ± 0.51	3.27 ± 0.71	4.19 ± 0.38	4.54 ± 0.13	2.04 ± 0.36	2.50 ± 0.32	3.12 ± 0.33	3.59 ± 0.41
	A	1.06 ± 0.14	0.94 ± 0.23	1.11 ± 0.15	1.13 ± 0.27	0.80 ± 0.20	0.75 ± 0.22	0.77 ± 0.19	0.68 ± 0.12
	E	1.80 ± 0.45	2.64 ± 0.44	2.29 ± 0.38	2.86 ± 0.78	1.18 ± 0.31	1.78 ± 0.42	1.49 ± 0.27	1.99 ± 0.50
	G	1.11 ± 0.23	0.90 ± 0.08	1.03 ± 0.21	1.04 ± 0.22	0.66 ± 0.08	0.60 ± 0.02	0.77 ± 0.17	0.65 ± 0.13
	J	0.77 ± 0.16	0.86 ± 0.09	1.02 ± 0.11	1.04 ± 0.20	0.52 ± 0.10	0.69 ± 0.12	0.74 ± 0.09	0.66 ± 0.13
	K	1.39 ± 0.31	2.56 ± 0.12	2.87 ± 0.61	2.50 ± 0.58	1.00 ± 0.23	1.80 ± 0.27	2.27 ± 0.31	1.24 ± 0.20
	M	1.83 ± 0.42	2.58 ± 0.29	2.84 ± 0.14	2.76 ± 0.66	1.26 ± 0.34	1.73 ± 0.25	2.01 ± 0.11	1.87 ± 0.44
	O	1.76 ± 0.40	2.91 ± 0.69	2.87 ± 0.15	2.73 ± 0.27	1.42 ± 0.34	2.12 ± 0.47	2.33 ± 0.11	1.94 ± 0.15
Pound cake	Control	5.86 ± 0.15	8.24 ± 1.36	8.28 ± 1.29	9.54 ± 1.33	5.49 ± 0.22	7.75 ± 1.11	7.50 ± 1.19	8.49 ± 1.16
	DATEM	6.58 ± 0.57	7.84 ± 1.22	9.14 ± 1.58	10.23 ± 2.05	6.13 ± 0.63	7.32 ± 1.27	8.31 ± 1.40	9.24 ± 1.98
	A	6.61 ± 0.67	6.89 ± 0.10	6.79 ± 0.49	7.34 ± 0.91	5.98 ± 0.36	6.42 ± 0.19	6.02 ± 0.48	6.47 ± 0.98
	E	4.94 ± 0.39	6.19 ± 0.78	5.74 ± 0.82	7.85 ± 1.05	4.56 ± 0.40	5.56 ± 0.72	5.07 ± 0.79	6.69 ± 0.99
	G	5.36 ± 0.45	6.53 ± 0.61	5.46 ± 0.37	6.73 ± 0.58	4.62 ± 0.50	5.89 ± 0.60	4.84 ± 0.35	5.75 ± 0.48
	J	4.77 ± 0.27	6.35 ± 0.83	5.77 ± 0.44	6.68 ± 0.88	4.20 ± 0.32	5.66 ± 0.79	5.04 ± 0.39	5.65 ± 0.73
	K	4.41 ± 0.72	7.26 ± 1.23	6.71 ± 0.91	7.90 ± 0.96	4.02 ± 0.67	6.54 ± 1.07	5.98 ± 0.88	7.04 ± 0.89
	M	4.41 ± 0.37	7.05 ± 0.90	6.10 ± 0.58	7.10 ± 1.13	4.01 ± 0.21	6.37 ± 0.75	5.29 ± 0.78	6.15 ± 0.87
	O	4.18 ± 0.47	6.61 ± 0.29	6.27 ± 1.07	7.04 ± 1.05	3.89 ± 0.47	5.98 ± 0.25	5.54 ± 1.08	6.07 ± 0.97
Brioche	Control	4.33 ± 0.17	6.58 ± 0.34	8.51 ± 0.52	10.73 ± 0.40	3.65 ± 0.41	5.53 ± 0.11	7.81 ± 0.35	9.69 ± 0.44
	DATEM	5.28 ± 0.62	7.59 ± 1.51	10.14 ± 1.04	12.68 ± 1.18	4.28 ± 0.25	6.58 ± 0.94	9.39 ± 0.80	11.51 ± 1.30
	A	4.33 ± 0.32	6.39 ± 0.61	7.81 ± 0.15	10.89 ± 1.06	3.26 ± 0.30	5.64 ± 0.56	7.17 ± 0.13	9.82 ± 1.03
	E	4.73 ± 0.47	6.46 ± 0.70	8.66 ± 0.86	11.85 ± 2.00	3.83 ± 0.37	6.13 ± 0.84	7.70 ± 0.61	10.57 ± 1.65
	G	4.49 ± 0.58	5.77 ± 1.01	8.12 ± 1.80	10.32 ± 1.50	3.48 ± 0.30	5.19 ± 0.98	7.48 ± 1.70	9.54 ± 1.68
	J	4.12 ± 0.42	5.51 ± 0.32	7.44 ± 1.35	9.91 ± 1.76	3.05 ± 0.33	4.93 ± 0.56	6.94 ± 1.30	9.05 ± 1.71
	K	3.84 ± 0.27	6.45 ± 0.80	8.14 ± 1.54	10.42 ± 2.23	3.28 ± 0.09	5.95 ± 1.03	7.22 ± 1.50	9.50 ± 2.00
	M	4.18 ± 0.64	6.70 ± 1.17	8.17 ± 1.54	10.66 ± 2.65	3.25 ± 0.44	6.13 ± 1.00	7.70 ± 1.55	10.16 ± 2.43
	O	4.06 ± 0.43	7.05 ± 0.27	8.78 ± 0.41	11.07 ± 1.05	3.05 ± 0.32	6.18 ± 0.43	8.11 ± 0.42	9.96 ± 0.70

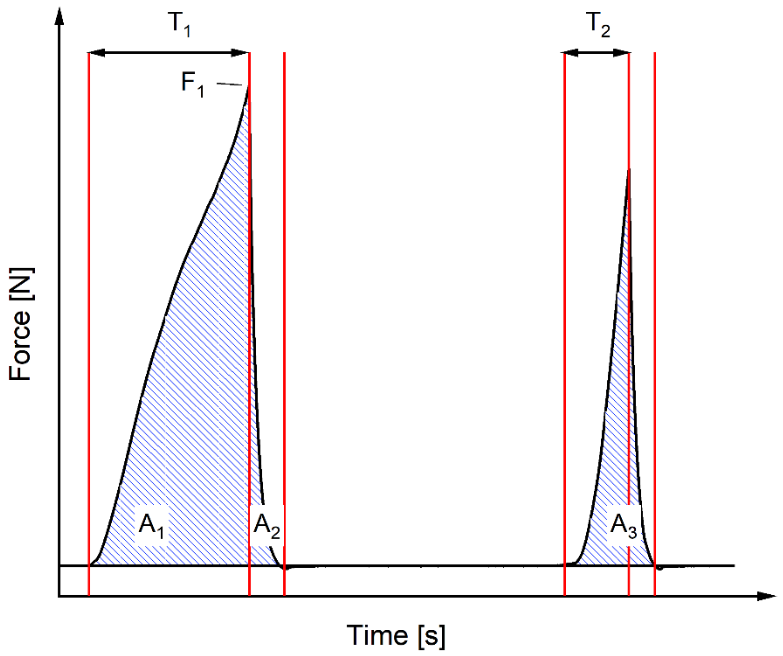

Textural property	Calculation
Firmness	$= F_1$
Springiness	$= T_1 / T_2$
Cohesiveness	$= (A_1 + A_2)/A_3$
Resilience	$= A_1/A_2$
Gumminess	= Firmness × Cohesiveness
Chewiness	= Gumminess × Springiness

Figure S1. Example of a texture profile analysis curve of cake (left) and calculation of textural properties (right). T_1 – time needed for the first compression; T_2 – time needed for the second compression; F_1 – maximum force of first compression; A_1 – area before the maximum peak height of first peak; A_2 – area after the maximum peak height of first peak; A_3 – area of the second peak.

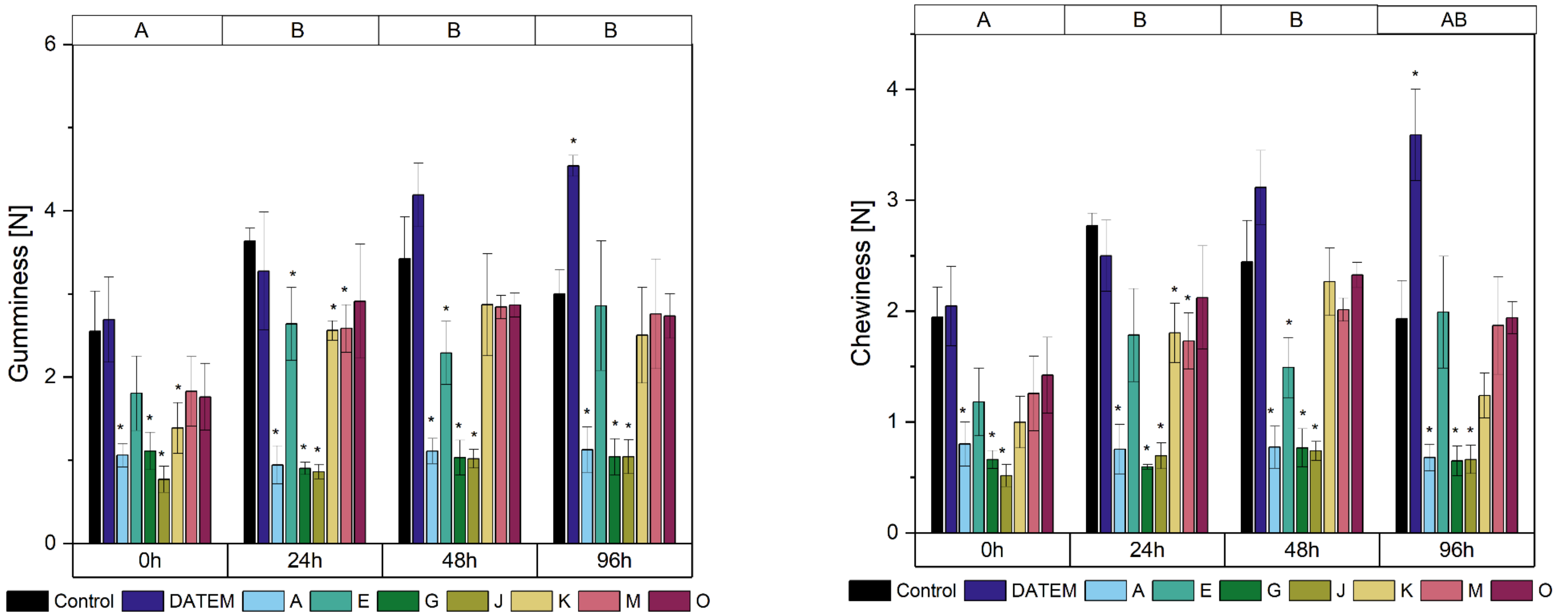

Figure S2. **Gumminess and chewiness** after 0 h, 24 h, 48 h and 96 h of differently modified **basic cake** samples (Control: sample without lipase addition, DATEM: sample with addition of DATEM, A-O: samples with addition of the respective lipase). Asterisks show a significant difference to the control sample of the respective time as calculated by ANOVA with Dunnett's test ($p \leq 0.05$, $n = 6$). Capital letters on the top indicate significant differences between means of all values of a certain time as calculated by ANOVA with Tukey's test ($p \leq 0.05$, $n = 6$).

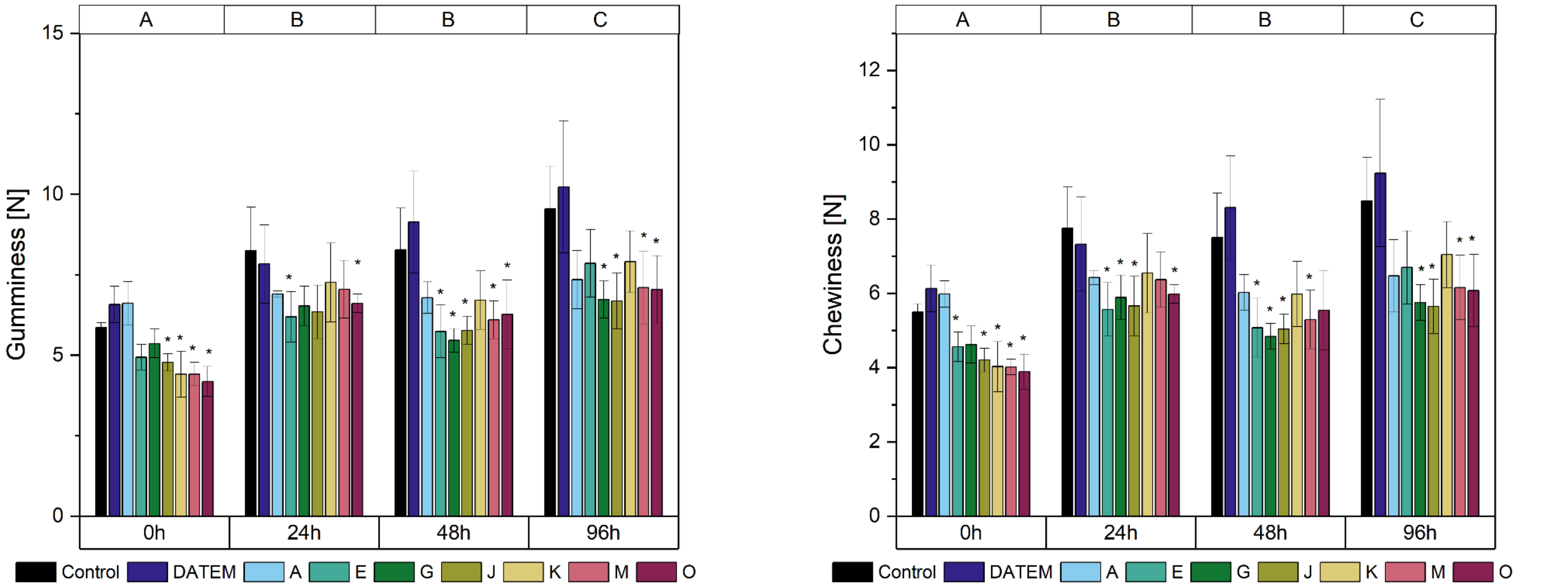

Figure S3. **Gumminess and chewiness** after 0 h, 24 h, 48 h and 96 h of differently modified **pound cake** samples (Control: sample without lipase addition, DATEM: sample with addition of DATEM, A-O: samples with addition of the respective lipase). Asterisks show a significant difference to the control sample of the respective time as calculated by ANOVA with Dunnett's test ($p \leq 0.05$, $n = 6$). Capital letters on the top indicate significant differences between means of all values of a certain time as calculated by ANOVA with Tukey's test ($p \leq 0.05$, $n = 6$).

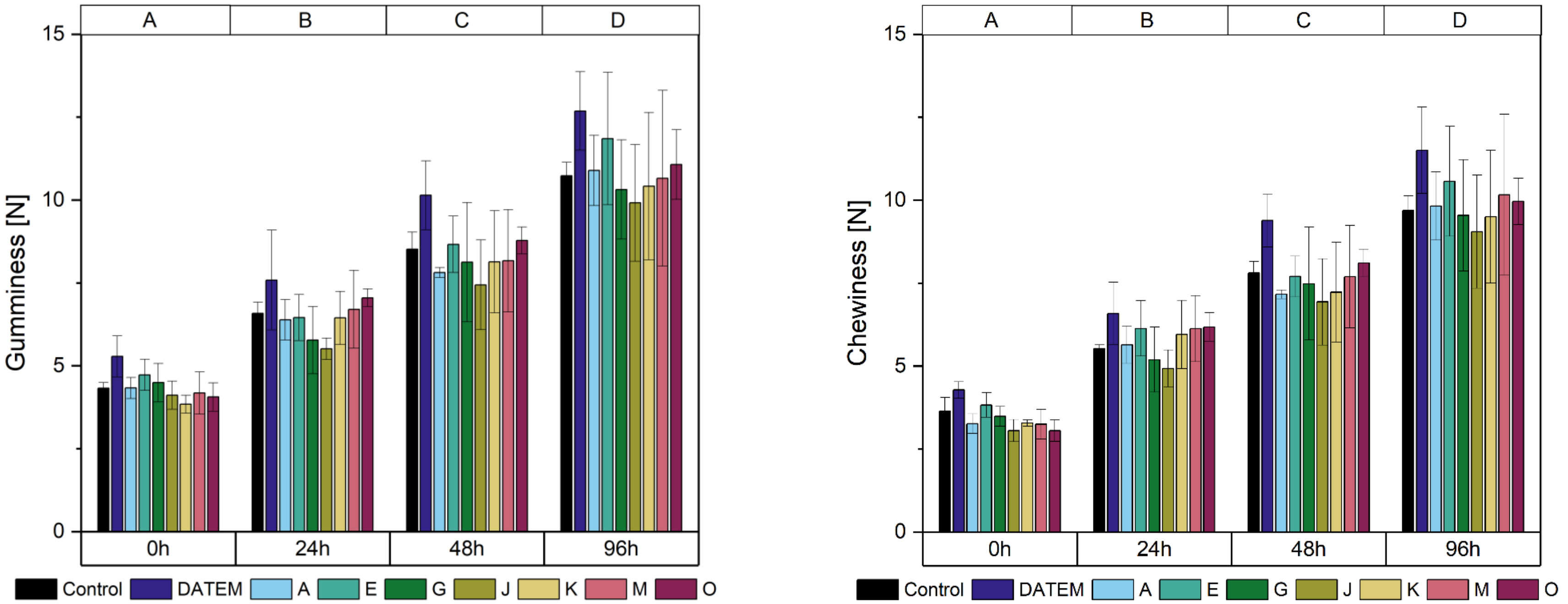

Figure S4. **Gumminess and chewiness** after 0 h, 24 h, 48 h and 96 h of differently modified **brioche** samples (Control: sample without lipase addition, DATEM: sample with addition of DATEM, A-O: samples with addition of the respective lipase). Asterisks show a significant difference to the control sample of the respective time as calculated by ANOVA with Dunnett's test ($p \leq 0.05$, $n = 6$). Capital letters on the top indicate significant differences between means of all values of a certain time as calculated by ANOVA with Tukey's test ($p \leq 0.05$, $n = 6$).
